# Altered Biogenesis and MicroRNA Content of Hippocampal Exosomes Following Experimental Status Epilepticus

**DOI:** 10.3389/fnins.2019.01404

**Published:** 2020-01-17

**Authors:** Aasia Batool, Thomas D. M. Hill, Ngoc T. Nguyen, Elena Langa, Mairéad Diviney, Catherine Mooney, Gary P. Brennan, Niamh M. C. Connolly, Amaya Sanz-Rodriguez, Brenton L. Cavanagh, David C. Henshall

**Affiliations:** ^1^Department of Physiology and Medical Physics, Royal College of Surgeons in Ireland, Dublin, Ireland; ^2^FutureNeuro SFI Research Centre, Royal College of Surgeons in Ireland, Dublin, Ireland; ^3^School of Computer Science, University College Dublin, Dublin, Ireland; ^4^Cellular and Molecular Imaging Core, Royal College of Surgeons in Ireland, Dublin, Ireland

**Keywords:** epileptogenesis, extracellular vesicle, neuroinflammation, non-coding RNA, seizure, temporal lobe epilepsy

## Abstract

Repetitive or prolonged seizures (status epilepticus) can damage neurons within the hippocampus, trigger gliosis, and generate an enduring state of hyperexcitability. Recent studies have suggested that microvesicles including exosomes are released from brain cells following stimulation and tissue injury, conveying contents between cells including microRNAs (miRNAs). Here, we characterized the effects of experimental status epilepticus on the expression of exosome biosynthesis components and analyzed miRNA content in exosome-enriched fractions. Status epilepticus induced by unilateral intra-amygdala kainic acid in mice resulted in acute subfield-specific, bi-directional changes in hippocampal transcripts associated with exosome biosynthesis including up-regulation of endosomal sorting complexes required for transport (ESCRT)-dependent and -independent pathways. Increased expression of exosome components including Alix were detectable in samples obtained 2 weeks after status epilepticus and changes occurred in both the ipsilateral and contralateral hippocampus. RNA sequencing of exosome-enriched fractions prepared using two different techniques detected a rich diversity of conserved miRNAs and showed that status epilepticus selectively alters miRNA contents. We also characterized editing sites of the exosome-enriched miRNAs and found six exosome-enriched miRNAs that were adenosine-to-inosine (ADAR) edited with the majority of the editing events predicted to occur within miRNA seed regions. However, the prevalence of these editing events was not altered by status epilepticus. These studies demonstrate that status epilepticus alters the exosome pathway and its miRNA content, but not editing patterns. Further functional studies will be needed to determine if these changes have pathophysiological significance for epileptogenesis.

## Highlights

- First study to specifically analyze exosome pathway in experimental epilepsy.
- Small RNA sequencing catalog defines microRNAs within hippocampus-derived exosomes.
- Select changes in exosomal microRNAs identified in experimental epilepsy.
- Select editing of microRNAs found within exosomes from the mouse hippocampus.

## Introduction

Prolonged seizures (status epilepticus) in humans are associated with significant morbidity and mortality (Betjemann and Lowenstein, 2015). Experimental modeling of status epilepticus, for example by systemic or focal application of chemoconvulsants such as kainic acid (KA), can reproduce hallmark pathophysiology. This includes select neuron loss, gliosis, and network reorganization within the hippocampus, and an enduring state of hyperexcitability characterized by recurrent spontaneous seizures (Henshall, 2017; Becker, 2018). Understanding the cell and molecular mechanisms that influence injury and repair processes after status epilepticus may lead to novel approaches for neuroprotection and anti-epileptogenesis.

An important role has emerged for microRNAs (miRNAs) in the post-transcriptional control of gene expression (Bartel, 2018). These short non-coding RNAs target complementary sequences within messenger RNAs (mRNA) leading to a reduction in mRNA stability or translation inhibition. Both human studies and animal models indicate that miRNAs may be master-regulators of the gene expression landscape following status epilepticus, and novel targets for seizure control or anti-epileptogenesis (Henshall et al., 2016). The mechanisms controlling the dysregulation of miRNAs in epilepsy remain poorly understood. DNA methylation and other epigenetic mechanisms have been found to influence the expression of a subset of miRNAs in experimental models and human studies (Miller-Delaney et al., 2015; Brennan et al., 2016). Recently, post-transcriptional modifications of miRNAs such as adenosine-to-inosine (A-to-I or ADAR) editing have been discovered to affect miRNA stability and subsequent availability, or to alter the targets of miRNAs (Li et al., 2018), including in the brain (Alon et al., 2012; Li et al., 2018).

It is likely that other mechanisms that control miRNA expression and function influence the pathogenesis of epilepsy. Emerging evidence suggests that miRNAs are loaded into and released from cells in extracellular vesicles such as exosomes (Valadi et al., 2007; Zhang et al., 2019). Exosomes are produced from an endosomal sorting complexes required for transport (ESCRT)-dependent pathway involving Hrs, Tsg101, Alix, and Stam1, and/or an ESCRT-independent pathway (Thery et al., 2002; Colombo et al., 2014) involving the function of phospholipase D2 (PLD2) and tetraspanins such as CD63 and CD81. Exosomes have been proposed to regulate neuron–glia communication and can be released from most major cell types of the brain in an activity or injury-dependent manner, including from astrocytes (Faure et al., 2006; Taylor et al., 2007), microglia (Potolicchio et al., 2005), and neurons (Faure et al., 2006; Lachenal et al., 2011). Studies have detected miRNAs within exosomes and suggested this as a novel mechanism of local and distant cell-to-cell communication (Valadi et al., 2007; Mittelbrunn et al., 2011). However, researchers have also questioned whether miRNA copy number within such vesicles is sufficient to produce biologically meaningful effects in recipient cells (Chevillet et al., 2014).

Little is known about the role or function of exosomal miRNAs and their editing in epilepsy. Altered expression of miRNAs has been reported within exosomes recovered from blood samples in patients with temporal lobe epilepsy (Yan et al., 2017) and dysregulation of miRNA editing has been reported in exosomes in non-CNS disease (Nigita et al., 2018). We recently found that cerebrospinal fluid from patients who experienced status epilepticus displayed different exosome miRNA content compared to other neurological diseases (Raoof et al., 2017). Functional studies suggest exosomes from human bone marrow-derived mesenchymal stem cells may regulate a neuro-inflammatory component in epilepsy (Long et al., 2017).

The role of the exosome pathway, the presence of miRNAs, and their editing within these extracellular vesicles remain poorly understood in the setting of status epilepticus. Here, we report the effects of experimental status epilepticus on the exosome biogenesis pathway and use small RNA sequencing to define their miRNA content and identify the editing landscape.

## Materials and Methods

### Animals

All procedures were performed in accordance with the principles of the European Union Directive (2010/63/EU) and were reviewed and approved by the Research Ethics Committee of the Royal College of Surgeons in Ireland (REC #842 and #1133) under license from the Health Products Regulatory Authority (AE19127/I084, AE19127/I089, and AE19127/I152), Dublin, Ireland. All efforts were made to minimize animal suffering and the number of animals used. Mice used in these experiments were 6–9 weeks old male C57BL/6OlaHsd from Envigo UK (formerly Harlan UK Ltd.), maintained at the Biomedical Research Facility, RCSI on a 12 h light–dark cycle at 21–23°C and humidity of 45–65% with *ad libitum* access to food and water.

### Status Epilepticus Model

Induction of status epilepticus was achieved via microinjection of KA (Sigma–Aldrich) into the right amygdala, propagating seizures to the hippocampus through the perforant pathway. As previously described (Mouri et al., 2008), mice were anesthetized using 3–5% isoflurane in oxygen and placed in a mouse-adapted stereotaxic frame. After a midline scalp incision, three cortical electrodes for electroencephalogram (EEG) recording were fixed with dental cement above the hippocampi and the frontal cortex. A guide cannula (coordinates from Bregma; AP = −0.95 mm, *L* = −2.85 mm) was fixed in place using dental cement. Mice were then placed in an incubator to recover. An injection cannula was then inserted through the guide for injection of either 0.3 μg KA or phosphate-buffered saline (PBS, for control mice) in 0.2 μL volume into the basolateral amygdala. This led to seizure onset within 5–10 min and after 40 min, mice were given an anti-convulsant (midazolam, 8 mg/kg; intraperitoneal) to curtail seizures and reduce morbidity and mortality (Diviney et al., 2015). The animals were then placed in a warm recovery chamber. Control and KA mice were killed at 4, 8, and 24 h, or 2 weeks. For euthanasia, mice were deeply anesthetized and transcardially perfused with PBS to remove blood components. For analysis of hippocampal subfields, the hippocampus was microdissected to yield individual CA1, CA3, and dentate gyrus (DG)-enriched subfields (Jimenez-Mateos et al., 2011).

### RNA Extraction and Real-Time PCR

Total RNA was extracted from microdissected subfields using the Trizol (Qiagen) method with chloroform-mediated phase separation and isopropanol-mediated precipitation. For analysis of transcripts, complementary DNA (cDNA) was produced from 1 μg of the total RNA by reverse transcription using Superscript III Reverse Transcriptase enzyme (Invitrogen). Quantitative PCR was performed using a LightCycler 1.5 (Roche Diagnostics) and QuantiTech SYBR Green PCR kit (Qiagen) as per the manufacturer’s instructions and 25 μM of primer mix was used. Specific primers for each gene assayed were purchased from Sigma and sequences used were: *Alix* F-ttcctgcaaaccgagttcct, R-acggcatattgtactggcca; *Arc* F-agcagcagacctgacatcct, R-gtgatgccctttccagacat; *Bok* F-ccaaaccc attcctttgtgg, R-gcctgggaaatcgagtgaaa; *CD63* F-tgtgggctgtgg gaatgatt; R-atgaaaagaccaaacgcccc; *CD81* F-tggttgcgtcatgatccaca, R-acaaggcaggtgaagaacgt; *Desmoplakin* F-tgctgccttttctgatacgc, R-gaaattcggagaagggatgc; *Hrs* F-acagcacatccaaaggcaga, R-ttccg tgcttcctcctgttt; *Pld2* F-tagccactgttgatgcccaa, R-tagccactgttg atgcccaa; *Rab27A* F-atgcctttcatcccagcact, R-tcacacacattaagccccgt; *Rab27b* F-agggaagtcaatgaacggca, R-ttctgctggcttttccccat; *Stam1* F-acaagcaaaagcaagcccag, R-ttcgtgctggtggttagtga; *TSG101* F-acccaccatacacagcaaca, R-agcttgttgtggcagggtat; and β*-actin* F-gggtgtgatggtgggaatgg, R-ggttggccttagggttcagg. β*-Actin* was used for the normalization of mRNA expression levels. Non-reverse transcribed extracts were used as negative controls. Relative mRNA transcript levels were assessed using the standard ^ΔΔ^CT method (Livak and Schmittgen, 2001). PCR data are presented as means ± standard error of mean (SEM). Two group comparisons were made using unpaired two-tailed Student’s *t*-test. Multi group comparisons were made using one-way ANOVA (Bonferroni’s correction). Significance was accepted at *p* < 0.05. Graphpad software was used for statistical analysis and for generating graphs which were further adjusted using Canvas software.

### Western Blotting

Briefly, extracted proteins were separated on SDS-PAGE gels and transferred to nitrocellulose membranes. Membranes were blocked for 1 h with 5% milk incubated with the primary antibodies against the following: Alix (Bethyl Laboratories, A302-938A), Calretinin (Swant, CG1), CD63 (Santa Cruz Biotechnology, SC-15363), Flotillin1 (Abcam, AB41927), Porin (Calbiochem, 529536), Rab27a, TSG101 (Genetex, GTX70255), β-Actin (Sigma–Aldrich, A5441) overnight at 4°C, and finally incubated with horseradish peroxidase-conjugated secondary antibodies (Cell Signaling Technology). Protein bands were visualized using SuperSignal^®^ West Pico Chemiluminescent Substrate (Millipore) and imaged using a Fuji-film LAS-3000/4000. Band densities were analyzed using ImageJ software. Protein levels were corrected to β-actin. Protein data were analyzed and presented as for PCR data (see above).

### Exosome Enriched Fractions – Ultracentrifugation Method

The protocol was adapted from Perez-Gonzalez et al. (2012). Mouse brain tissue or hippocampi were removed from storage (−80°C) and placed in 1 ml of Hibernate A (Biosciences) and cut into small pieces. An additional 8 ml of Hibernate A was added and using a 10 ml plastic pipette, tissue was loosened further and manually triturated to obtain tissue dissociation. The solution was centrifuged at 300 × *g* for 10 min; note that this and further centrifugations were carried at 4°C. A distinct pellet and supernatant were observed. The supernatant was passed through a 40-μm mesh filter. The filtrate was passed through a 0.2-μm syringe filter. The filtrate was then centrifuged at 2000 × *g* for 10 min. Ice-cold PBS was added and centrifuged at 10,000 × *g* for 30 min. The supernatant was pipetted to an ultracentrifuge tube (polyalamar, thin-walled tubes, Fisher Scientific) which was ultracentrifuged at 100,000 × *g* (23,200 rpm on surespin360 rotor) for 70 min to pellet exosome-enriched fractions (EEFs). The tube was inverted and EEFs re-suspended in PBS.

### Exosome Enriched Fractions – Exosome Precipitation Solution (Kit) Method

Exosome-enriched fractions were also prepared using a kit-based method. We used the adapted exosome isolation protocol from above but omitted the 100,000 × *g* ultracentrifugation (UC) step. Briefly, 1 ml of cold PBS was used to cut brain tissue into smaller pieces and then was manually triturated with additional 1 ml PBS. The solutions were put through low speed centrifugations and also passed through two filters as above. Without additional PBS, after the 10,000 × *g* centrifugation, the exosome precipitation solution provided in the ExoQuick kit for biofluids was used, combining 500 μl sample with 120 μl kit solution followed by mixing at 4°C for 30 min. The sample was then spun at 13,000 rpm for 2 min at 4°C and then re-suspended in PBS.

### Transmission Electron Microscopy

For immunogold staining of exosomes, a nickel grid was placed on 5 μl of isolate for 45 min and fixed in 2% glutaraldehyde. Non-specific binding was blocked using 0.5% bovine serum albumin (BSA) in PBS for 15 min at RT, incubated with primary antibody at 1:50 for 60 min at RT, washed 3 × 3 min in PBS, incubated with gold conjugated secondary for 45 min at room temperature, washed with dH_2_O 3 × 3 min, and blotted using filter paper before allowing to dry completely. Imaging was performed with a Hitachi H7650 transmission electron microscope at an accelerating voltage of 100 kV.

### Zetasizer

A Zetasizer Nano ZS –1.0 was used to measure the size of particles in the EEF suspension. It was ensured that the sample was visibly a uniform homogenous mixture suspended in a volume of 850–1000 μl PBS. The disposable capillary cell (DTS1070) used was washed with PBS to ensure no residues from earlier samples were left. The Zeta cell was stoppered and then inserted into the instrument for particle size measuring via dynamic light scattering.

### Small RNA Library Preparation and RNA-Sequencing

RNA sequencing was performed to profile miRNAs in hippocampus-derived EEFs. A pilot study was performed to determine the minimum amount of hippocampal starting material needed to generate sequencing libraries from EEFs. The pilot study confirmed the kit-based technique required fewer hippocampi. Therefore, *n* = 5 hippocampi were pooled for each sample in the UC-based technique and *n* = 4 hippocampi were pooled for each sample in the kit-based technique. Three biological replicates of pooled samples obtained at 24 h and 2 week time-points were performed using the UC- and kit-based methods, separately. RNA was extracted from the EEFs post RNase-treatment. Isolated exosomes, re-suspended in 100 μl PBS, were treated with 2 μl (20 μg) RNase-A (Fisher Scientific). This was incubated at 37°C for 10 min on a shaker. To stop the reaction, 20 μl (400 U) RNase Inhibitor (Biosciences) was added and this was incubated at 37°C for 20 min. RNA extraction was carried out similar to above with 800 μl Trizol LS (Qiagen) used to triturate the sample, followed by a chloroform-mediated phase separation and isopropanol-mediated precipitation. The RNA was reconstituted in 7–9 μl RNase-free water at 60°C for 10 min, shaking at 800 rpm. After extraction of RNA, 5 μl from each sample was used to construct small cDNA libraries using the Illumina TruSeq Small RNA library kit, with each sample barcoded for identification. Small amounts of RNA input required slight modification in the protocol whereby all kit reagents were halved in order to prevent substantial adapter dimer formation. Libraries were size-selected using a Pippin Prep (Sage Science) with 3% agarose dye-free cassettes and size selection was validated using a 2100 High Sensitivity DNA Bioanalyzer chip (Agilent 2100). The concentration of each library was determined using the HS-dsDNA kit for Qubit. The cDNA libraries already barcoded for identification of each sample were pooled and sequenced at the Trinseq Facility at the Institute for Molecular Medicine at St. James Hospital, Dublin, on an Illumina miSeq (SY-410-1003).

### RNA-Sequencing Data Processing and Analysis

Sequencing data were uploaded to the Chimira webserver (Vitsios and Enright, 2015) where the sequences were adapter trimmed and mapped against miRBase v21 hairpin sequences to generate count-based miRNA expression data (Kozomara and Griffiths-Jones, 2014). Sequencing data are available from the Gene Expression Omnibus (*GSE136695*). MiRNA differential expression analyses were performed using R/Bioconductor. Normalization of miRNA expression count was by the method of trimmed mean of *M*-values (TMM) (Robinson and Oshlack, 2010). EdgeR (Robinson et al., 2010) and Limma (Ritchie et al., 2015) packages were utilized following the protocol by Law et al. (2016). An miRNA was considered to be differentially expressed if the *p*-value was <0.05. These were non-adjusted for multiple comparisons, except where indicated for individual miRNA validations in UC samples. Taqman-specific miRNA assays were carried out for individual miRNAs as a validation step, as per manufacturer’s guidelines. Further, likely cellular origins of EEFs based on their miRNA content were explored using literature. Enrichment of cell type-specific miRNAs in brain EEFs was drawn from specific papers (Jovicic et al., 2013; Butovsky et al., 2014; Ludwig et al., 2016). MiRNA were categorized based on strong association with neurons, astrocytes, or microglial origin. Furthermore, comparisons were drawn between data obtained here and the available EV database, i.e., ExoCarta (Mathivanan and Simpson, 2009).

For miRNA target identification and Gene Ontology (GO) enrichment analysis, experimentally validated targets were retrieved from miRTarBase Release 7.0 (Chou et al., 2018), TarBase v.8 (Karagkouni et al., 2018), and miRecords (Xiao et al., 2009) while predicted targets were retrieved from TargetScan Release 7.2 (Agarwal et al., 2015) and miRDB Version 6.0 (Liu and Wang, 2019). We calculated a miRNA–target interaction (MTI) score based on combined prediction algorithm scores and the number of publications associated with the validated MTIs, as described previously (Raoof et al., 2018). Enrichment analysis of GO terms was performed on all MTIs with a score >0.1 using ReactomePA R/Bioconductor package (Yu and He, 2016). GO terms with enrichment adjusted *p*-value < 0.05 were considered significant.

For editing analysis, the count-based data of miRNA modifications were also generated from Chimira (Vitsios and Enright, 2015). The data were then processed using custom python and unix bash scripts separately for UC and kit samples. Briefly, only miRNAs with at least 10 read counts and modifications within these mature miRNAs (internal modifications) were considered in this analysis. For all reported editing positions, the read counts for each modification were calculated. Considering an expected sequencing error rate of 0.01 (which is equal to the base quality Phred score of 20), we applied the binomial cumulative distribution *B*(*n*, *p* = 0.01), where *n* is the total read count of an miRNA and *p* is the probability of observing a miscalled nucleotide different from the reference nucleotide at any reported positions of the miRNA to (1) exclude the modifications arising from potential sequencing errors and (2) to keep only modifications which are significantly (FDR < 0.05) over-represented (referred as edited sites) compared to the expected sequencing error rate. This approach for identifying editing sites has been described (Alon et al., 2012). Only edited sites identified in at least two UC or two kit samples per group were kept. Then, unpaired two-sided *t*-tests were used to identify the differences in editing levels (defined as the ratio of the read count of each non-reference modification to the total read count of the miRNA where the editing occurred) between the groups at two different time points. Editing sites that passed the FDR threshold of 0.05 were considered as differentially edited sites between groups. All statistical calculations were performed in R and all the codes were available at GitHub^1^. Sequence motifs of the edited sites then were generated on Weblogo (Crooks et al., 2004).

## Results

### Subfield-Specific Changes in Exosome Biogenesis Components After Status Epilepticus

To determine if prolonged seizures *in vivo* affect the exosome biogenesis pathway we analyzed the expression of a set of genes from the ESCRT-dependent and -independent pathways after status epilepticus. Prolonged seizures were induced by unilateral microinjection of KA into the amygdala of mice. Consistent with previous reports (Araki et al., 2002; Mouri et al., 2008; Diviney et al., 2015; Jimenez-Mateos et al., 2015), this produced characteristic damage to the ipsilateral hippocampus, comprising irreversible neuronal death confined mainly to the CA3 subfield (Supplementary Figure 1). Occasional cell death was evident within the ipsilateral hilar region of the DG and CA1 subfield while the contralateral hippocampus did not display irreversible neuronal death (Supplementary Figure 1). Brain tissue sections from mice 2 weeks after status epilepticus no longer displayed active neuronal death but featured characteristic neuron loss with the ipsilateral CA3 subfield and attendant astrogliosis.

**FIGURE 1:**
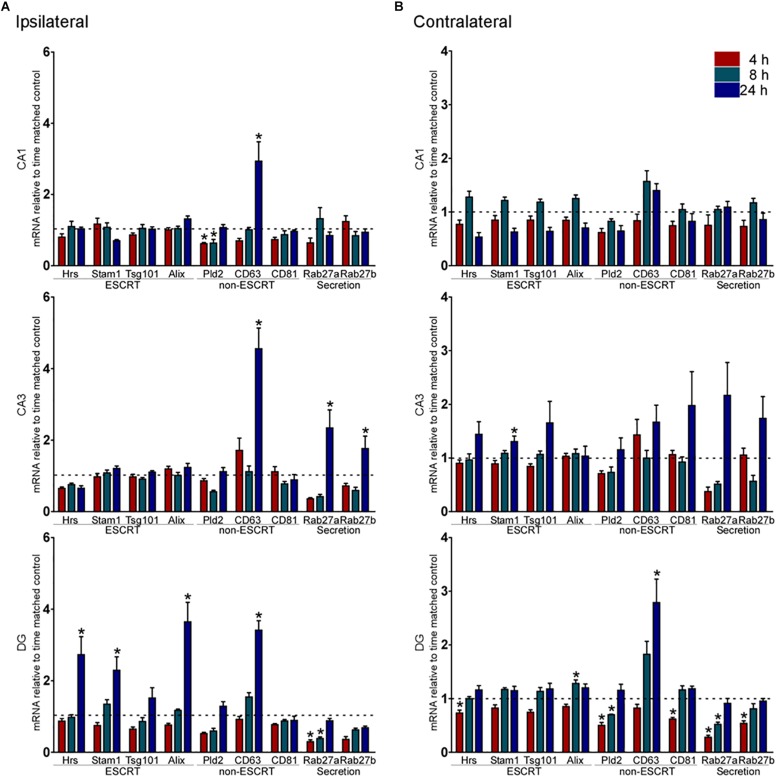
Acute changes to exosome biogenesis transcripts after status epilepticus in hippocampal subfields. Graphs show transcript (mRNA) levels 4, 8, and 24 h after status epilepticus (SE) induced by KA compared to control in each subfield of the **(A)** ipsilateral and **(B)** contralateral, hippocampus. Levels were normalized to β*-actin*. Shown are ESCRT-dependent exosome biogenesis transcripts, ESCRT-independent exosome biogenesis transcripts, and potential exosome secretion transcripts. Dotted line indicates control level. Graphs show mean ± SEM. ^∗^*p* < 0.05 compared to matching control. *n* = 4–6 per group; ANOVA with Bonferroni *post hoc* test.

Exosome biogenesis components were compared between KA and PBS injected-control samples for each major subfield at 4, 8, and 24 h (acute phase) or 2 weeks later (chronic). Analysis of the ESCRT-dependent genes in the ipsilateral CA1 and CA3 subfields of the hippocampus found no differences in expression of any pathway genes up to 24 h after status epilepticus (Figure 1A). In contrast, three ESCRT-dependent pathway genes (*Hrs*, *Stam1*, and *Alix*) were upregulated within the ipsilateral DG subfield at 24 h (Figure 1A). In the contralateral CA1 subfield, there were no significant changes in expression of ESCRT-dependent genes up to 24 h after status epilepticus (Figure 1B). There was upregulation of *Stam1* within the contralateral CA3 subfield at 24 h. Bi-directional changes were found for ESCRT-associated genes within the contralateral DG, with down-regulation of *Hrs* and upregulation of *Alix* (Figure 1B).

There were several changes to the expression of ESCRT-independent pathway transcripts after status epilepticus. Expression of *CD63* was increased in all three ipsilateral subfields at 24 h (Figure 1A). *Rab27a* and *Rab27b* expression was also increased after status epilepticus in the ipsilateral CA3 subfield (Figure 1A). In contrast, *Rab27a* levels were down-regulated within the ipsilateral DG. Levels of *Pld2* showed a decrease at earlier time-points in CA1 only. There were no changes in any ESCRT-independent genes tested in the contralateral CA1 or CA3 subfields, while the contralateral DG displayed bi-directional changes including lower levels of *Pld2*, *Rab27a*, and *Rab27b* (Figure 1B).

**FIGURE 2:**
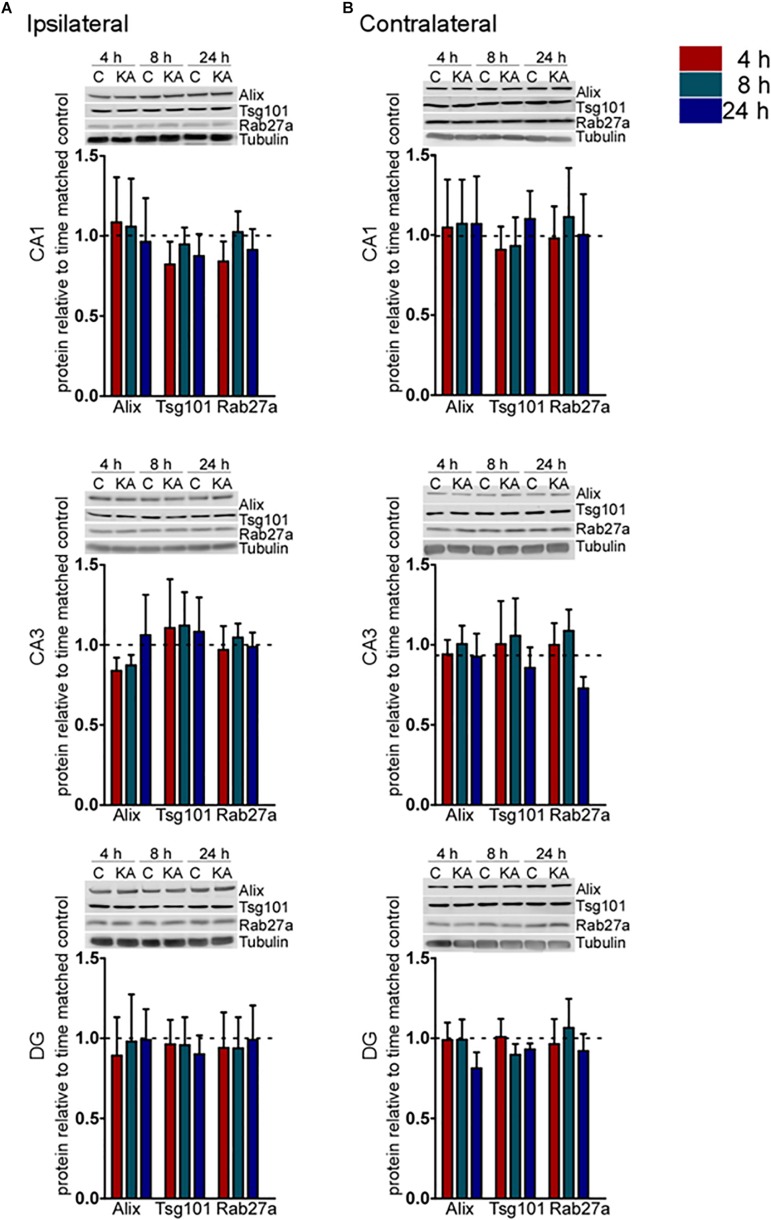
Acute regulation of protein levels of exosome biogenesis pathways after status epilepticus in hippocampal subfields. Representative immunoblots and graphs show protein levels of Alix, Tsg101, and Rab27a exosome pathway components 4, 8, and 24 h after SE compared to control (C) in each subfield of the **(A)** ipsilateral and **(B)** contralateral, hippocampus. Graphs show mean ± SEM. Protein levels were normalized to tubulin (*n* = 4–5 per group; ANOVA with Bonferroni *post hoc* test showed no significant differences).

To explore whether these transcriptional changes were associated with corresponding protein changes we performed immunoblotting for a selection of pathway components (Figure 2). Western blot analysis of hippocampal samples for Alix, Tsg101, and Rab27a showed no statistical difference (*p* > 0.05) in protein levels over the time course examined in either the ipsilateral or contralateral hippocampus (Figures 2A,B). These results suggest that changes to the expression of exosome pathways are restricted to a transcriptional level during the acute phase following status epilepticus. This is most evident within the DG subfield of the damaged ipsilateral hippocampus and also affects subfields that display neuron loss in the model.

### Subfield-Specific Changes to Exosome-Associated Pathway in the Hippocampus 2 Weeks Following Status Epilepticus

We next investigated whether status epilepticus causes any longer-lasting changes to the expression of exosome pathway components, assessing the same transcripts 2 weeks after status epilepticus when spontaneous recurrent seizures typically occur in this model (Mouri et al., 2008; Jimenez-Mateos et al., 2015). However, mice were not monitored electrographically or by video to determine whether epilepsy occurred.

**FIGURE 3:**
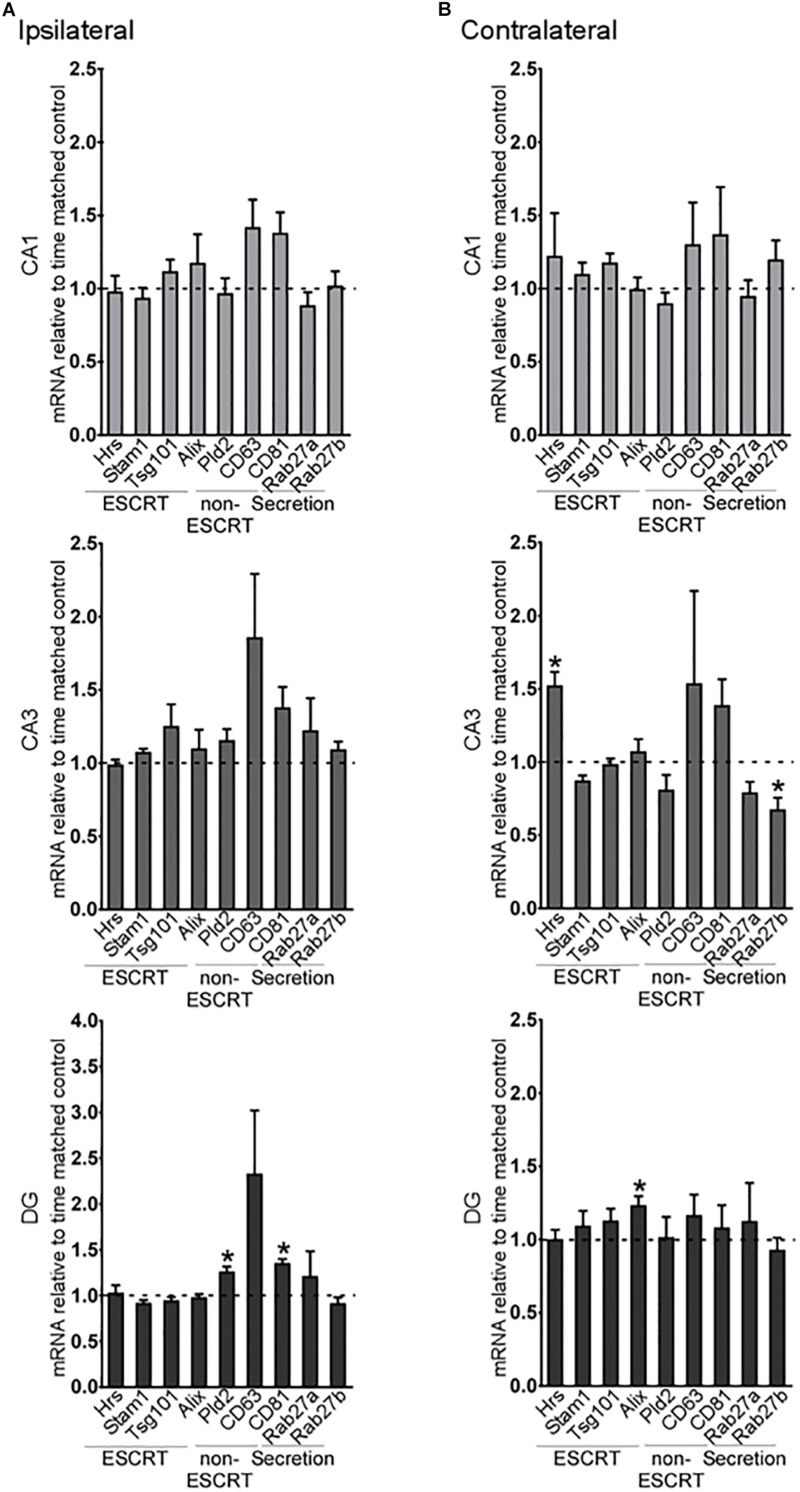
Long-term regulation of exosome biogenesis transcripts in hippocampal subfields after status epilepticus. Graphs show mRNA levels of exosome pathway components in samples obtained 2 weeks after SE for each subfield in the **(A)** ipsilateral and **(B)** contralateral hippocampus. Transcript levels were normalized to β*-actin*. Graphs show mean ± SEM. *n* = 5 per group. ^∗^*p* < 0.05, *t*-test comparing to control.

A small number of exosome biogenesis genes were found to be differentially expressed in the hippocampus of 2-week post-status epilepticus mice (Figure 3). On the ipsilateral side, there were no differences in expression of any of the exosome pathway transcripts in the CA1 or CA3 subfields (Figure 3A), but expression of two of the ESCRT-independent genes, *CD81* and *Pld2*, was increased in the DG. On the contralateral side, *Hrs* expression was increased in the CA3 region and *Rab27b* was decreased (Figure 3B). In the DG, only *Alix* levels were altered in the contralateral hippocampus (Figure 3B).

**FIGURE 4:**
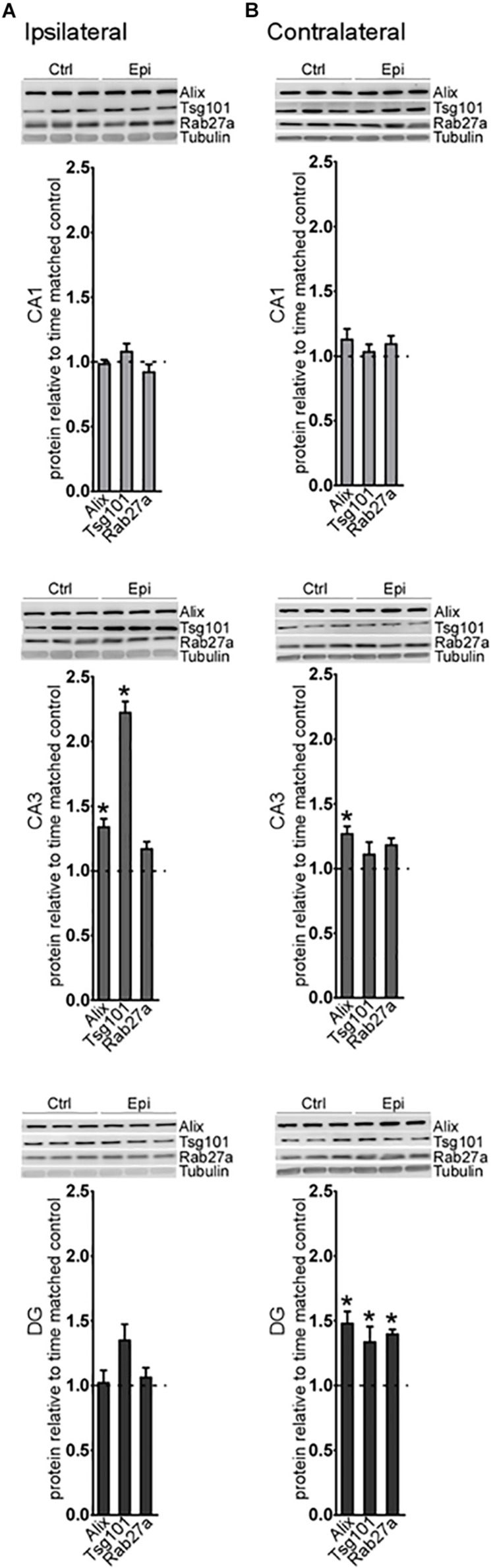
Long-term regulation of exosome biogenesis proteins after status epilepticus in hippocampal subfields. Representative western blots and graphs show levels of a set of exosome proteins in samples obtained 2 weeks after SE for each subfield in the **(A)** ipsilateral and **(B)** contralateral hippocampus. Protein levels are normalized to tubulin. Graphs show mean ± SEM. *n* = 5–6 per group. ^∗^*p* < 0.05, *t*-test comparing to control.

Analysis of a selection of the proteins at this time point found an increase in Alix and Tsg101 in the CA3 subfield on the ipsilateral hippocampus (Figure 4A). On the contralateral side, Alix was also increased in the CA3, while, in the DG, levels of Alix, Tsg101, and Rab27a were all elevated (Figure 4B). Thus, at later time points after status epilepticus there are fewer transcriptional changes in the exosome pathway but changes at the protein level become more apparent, suggestive of a shift toward post-transcriptional mechanisms of gene expression control. Taken together, these studies suggest modest spatio-temporal expression changes in the exosome biogenesis pathway after status epilepticus.

### Preparation of Exosome-Enriched Fractions From Mouse Hippocampus

In order to explore the miRNA content of mouse hippocampal exosomes, we used both kit- and centrifugation-based methods to obtain EEFs. Using a kit-based method, we confirmed EEFs were enriched for exosomal markers Alix and CD63 in control (naïve) mouse brain samples and had an appropriate size of approximately 100 nm (Supplementary Figure 2a). EEFs prepared from naïve brain samples using an UC-based technique were also enriched for appropriate markers including Alix and Flotillin1 and had an appropriate size and spherical morphology (Supplementary Figure 2b). Immuno-gold staining of EEF-prepared samples using UC confirmed CD63 on the exosomes from isolates (Supplementary Figure 2c). The two isolation techniques were also compared for the relative presence of contaminants, i.e., non-exosomal markers, namely porin (mitochondria marker) and calretinin [endoplasmic reticulum (ER) marker] (Supplementary Figure 2d). With the kit-derived EEFs we could detect calretinin but no mitochondrial contamination. In contrast, the UC-prepared EEFs showed the presence of the ER and mitochondrial markers but were more enriched for Flotillin1, a well-known exosome marker. Accordingly, we proceeded to undertake the miRNA analysis using samples prepared using both methods. Since surface-bound RNAs including miRNAs can be a major source of contamination in exosome studies, sequencing was performed after EEF samples were treated with RNase.

### Mouse Hippocampus-Derived EEFs Contain a Conserved Set of Abundant miRNAs

**FIGURE 5:**
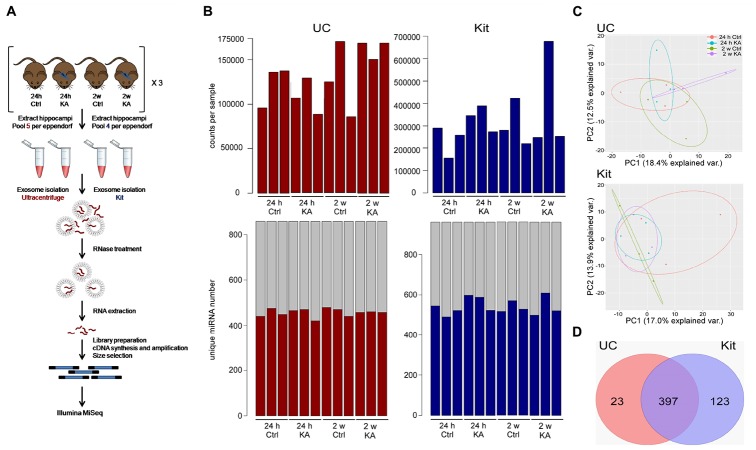
Study design and sequencing of EEF miRNAs. **(A)** Schematic of experimental design for miRNA sequencing. EEFs were prepared using hippocampi from sets of control (Ctrl) or KA-treated mice using UC- or kit-based methods. EEFs were then RNAse treated to remove non-enclosed miRNAs and processed for RNA sequencing. **(B) Top:** bar plot showing total number of counts per sample mapping to miRBase_V21. **Bottom:** Number of confidently called, unique miRNA identified in each sample. **(C)** Principal component analysis (PCA) plot showing clustering of the 420 and 520 filtered miRNAs from UC and kit samples, respectively. **(D)** Venn diagram shows the number of detected miRNAs in exosomes post filtration in UC and in kit, highlighting EEF miRNAs common to both methods.

Small RNA sequencing was performed on EEFs prepared from pools of whole hippocampi from mice subject to status epilepticus at two time points (24 h and 2 weeks) and time-matched controls. Figure 5A provides an overview of the sequencing. The total count numbers per sample as mapped to miRBase_V21 are highlighted (Figure 5B). The data were filtered to remove any low-quality sequences; a count per million (CPM) value of 1 (log-CPM of zero) in at least six samples was used as a threshold for miRNAs to be considered “expressed.”

A total of 420 and 520 unique miRNAs were detected in total across samples using the UC and kit methods, respectively (Figure 5B). A principal component analysis (PCA) of these miRNAs from the UC and kit samples showed low variance between samples using both methods (Figure 5C). This indicates a broadly similar miRNA composition between methods and between control and seizure samples. We found 397 miRNAs were common to both UC and kit-based preparation techniques (Figure 5D). Thus, EEFs contain diverse and largely consistent miRNA contents independent of preparation technique.

**FIGURE 6:**
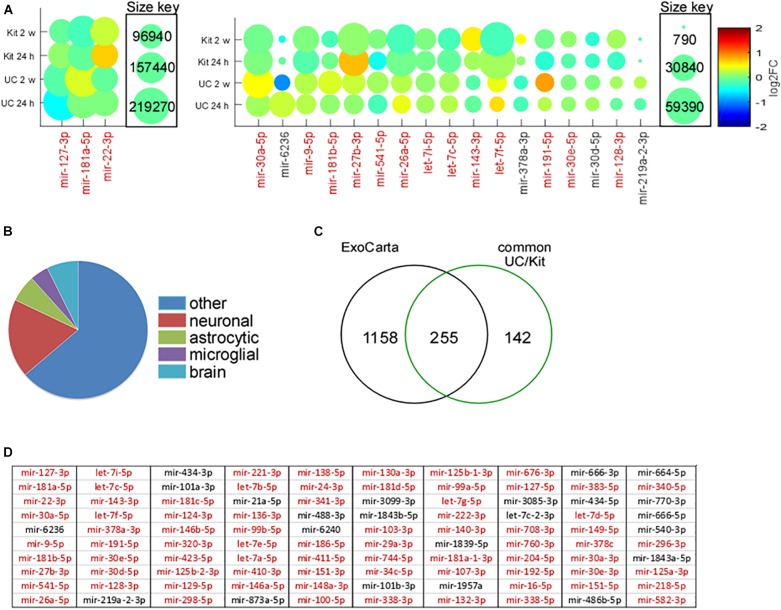
Abundance, cell origins, and ExoCarta analysis of EEF miRNA content. **(A)** Amounts of miRNAs identified by small RNA sequencing of EEFs from mouse hippocampus. MiRNAs are ordered on a Matlab-based visual graphic, according to average log2FC across all samples, from highest to lowest abundance. The size of the visual graphic bubble shows the number of reads (CPM) of each miRNA detected in the samples and its color represents the log2FC in KA relative to PBS control levels. Presented are the top 20 miRNA in the UC-derived EEFs, of which 16 are common across all 24 samples (in red). **(B)** Pie chart depicts the assignment of miRNA based on likely cell type for the common 397 miRNAs detected in both UC and kit EEFs. **(C)** Venn diagram showing overlap (∼64%; 255 of 397) of the detected miRNA with those listed in the exosome contents database, Exocarta. **(D)** Top 100 miRNA (most abundant based on average across all 12 samples in UC) expressed in EEFs from both UC and kit. The miRNAs colored red are listed in ExoCarta.

We next explored the most abundant miRNAs within EEFs, and how these were affected by status epilepticus (Figure 6). The three most abundant miRNA found in EEFs – 127-3p, miR-181a-5p, and miR-22-3p – were common to both isolation techniques in both control and KA samples, of which miR-127-3p is known to be exosome- and brain-enriched (Koppers-Lalic et al., 2014). Including these, 16 of the 20 most abundant miRNAs in EEFs across all samples were common: miR-30a-5p, miR-9-5p, miR-181b-5p, miR-27b-3p, miR-541-5p, miR-26a-5p, let-7i-5p, let-7c-5p, miR-143-3p, let-7f-5p, miR-191-5p, miR-30e-5p, and miR-128-3p (Figure 6A). This confirms high conservation of miRNA abundance within EEFs, regardless of isolation technique.

### miRNA Composition of Hippocampal EEFs Suggests Multiple Cellular Origins

We next explored the likely cellular origins of EEFs based on their miRNA content (Figure 6B). Attribution of a miRNA to a specific cell type was based on *in vitro* and *in vivo* datasets (Bak et al., 2008; Jovicic et al., 2013; Butovsky et al., 2014; Ludwig et al., 2016). Of the 397 miRNAs common to both EEF preparation methods, 73 were strongly associated with neuronal expression, 25 were known to be enriched in astrocytes, and 17 were of likely microglial origin (Figure 6B). Last, we compared our hippocampus EEF-miRNA content to a reference database (ExoCarta), a manually curated reference database of exosomal proteins, RNA, and lipids. This revealed that 255 out of the 397 (∼64%) of the commonly detected miRNA were listed in ExoCarta (Figure 6C), with strong overlap of the top 100 miRNA (Figure 6D).

### Temporal Changes to Exosome-Enriched Fraction miRNA Content After Status Epilepticus

**FIGURE 7:**
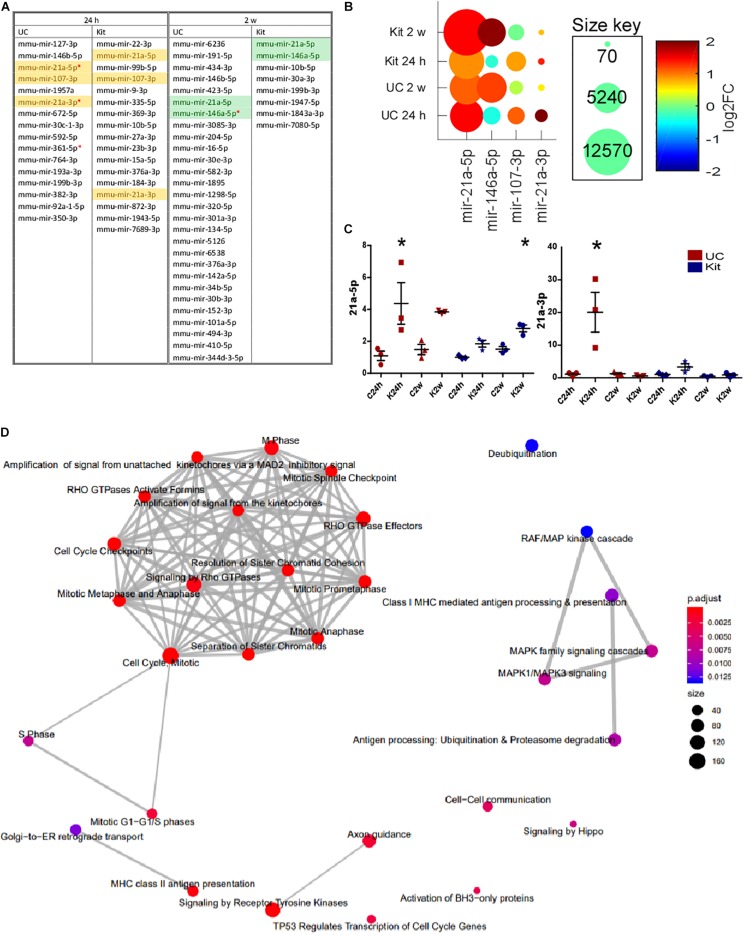
Differential expression of miRNA in EEFs after status epilepticus. **(A)** The table shows miRNA which were detected as differentially expressed between control and KA at 24 h and 2-weeks, at unadjusted *p*-value ≤ 0.05. Highlighted miRNAs are those differentially expressed in EEFs using both UC and kit methods. Red asterisk indicates miRNA which were differentially expressed at an adjusted *p*-value ≤ 0.05 in UC samples. **(B)** Conserved miRNA changes after status epilepticus. The graph shows levels of miR-21a-3p, miR-21a-5p, and miR-107-3p (increased at 24 h), and miR-21a-5p and miR-146a-5p (increased at 2-weeks). Of these, significance for miR-21a-3p and miR-21a-5p at 24 h and miR-146a-5p at 2-weeks was only found in the UC method at an adjusted *p*-value of <0.05. **(C)** Validation of sequencing results using individual miRNA assays confirming increased miR-21a-5p and -3p. UC, in red; kit, in blue. miRNA levels are normalized to average of 25-3p and 92b-3p. Graphs show mean ± SEM. ^∗^*p* < 0.05 comparing to matching control. *n* = 3 per group using the same samples as were sequenced (ANOVA with Bonferroni *post hoc* test). **(D)** Network map of GO terms significantly enriched among the targets of the four miRNAs (miR-21a-3p, miR-21a-5p, miR-107-3p, and miR-146a-5p). The network was generated using ReactomePA R/Bioconductor package. The node color indicates the significance of the enrichment (adjusted *p*-value) while the node size indicates the number of miRNA targets were found in that GO term category.

We investigated differential expression of all miRNAs in EEFs obtained either 24 h or 2 weeks following status epilepticus. Status epilepticus changed the EEF content for 16 and 17 miRNAs in UC or kit samples at 24 h, respectively (Figure 7A). In samples obtained 2 weeks after status epilepticus there were significant differences for 28 and 8 miRNAs in UC or kit samples, respectively. The identities of these miRNAs are provided in Figure 7A. This included, at 24 h after status epilepticus, changes to levels of miR-21a-3p, miR-21a-5p, and miR-107-3p. Levels of miR-21a-5p and miR-146a-5p were found to be differentially expressed in EEFs in the 2-week samples using either extraction method (Figure 7B).

As an additional step, we validated the RNA sequencing analysis of EEF miRNAs by running individual miRNA Taqman assays (Figure 7C). Validation of the results confirmed altered expression of miRNAs in EEFs prepared using the UC method only, including up-regulation of miR-21a-3p at the 24 h time-point after status epilepticus and miR-21a-5p at both 24 h and 2-week time-points. Taken together, these studies demonstrate that status epilepticus produces select changes to the miRNA content of EEFs.

### MiRNA Target Identification and Gene Ontology Enrichment Analysis

Then, we performed *in silico* identification of targets for these four miRNAs that are differentially expressed in exosome-enriched fractions after status epilepticus (miR-21a-3p, miR-21a-5p, miR-107-3p, and miR-146a-5p) since this analysis will shed some important insights into the role of these miRNAs. Experimentally validated as well as predicted targets were retrieved from several miRNA target databases and a MTI score was calculated for each interaction (as described in the section “Materials and Methods”). A total of 4,539 targets with MTI scores >0.1 were included in the GO enrichment analysis. Our analysis showed the miRNA targets were most significantly enriched (adjusted *p*-value < 0.05) among GO terms associated with the regulation of cell cycle and division (Figure 7D), including several genes encoding for cyclin protein family, cell division cycle proteins, and centromere protein family.

### Select A-to-I Editing in Exosome-Enriched Fraction miRNAs

**FIGURE 8:**
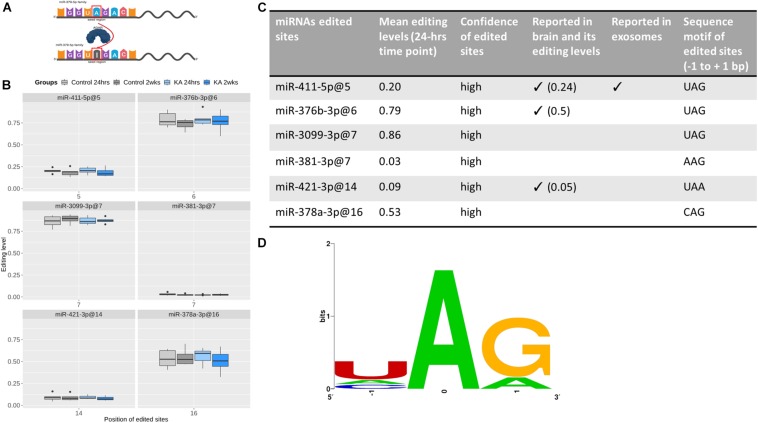
RNA editing of EEF-derived miRNA. **(A)** Graphic illustration of A-to-I editing, which is catalyzed by ADAR enzymes. miRNA editing can occur in seed regions (as shown here) or outside of the seed regions. This graph was created with BioRender. **(B)** Boxplots showing the editing levels (median with interquartile range), in control and KA samples at the two different time points for six edited sites detected in EEF miRNAs. **(C)** The table shows evidence supporting the edited sites and **(D)** sequence motifs of the edited sites. This shows the nucleotide preferences of all sequences surrounding the six edited sites [three-bases long, with the edited nucleotide Adenosine (A) is centered]. All the six edited sites have preferable sequence motifs for ADAR enzymes, which is (1) over-represented uridine (U) but depleted guanosine (G) at the nucleotides before the edited sites and (2) over-represented guanosine at the nucleotides after the edited sites. Confidence of edited site (Li et al., 2018) reported in brain (Alon et al., 2012) and reported in exosomes (Nigita et al., 2018).

Last, we explored potential miRNA editing events within the EEF samples. Only ADAR editing, in which ADAR enzymes catalyze the replacement of adenosine (A) by inosine (I) on double-strand RNAs, was detected in our data (Figure 8). To provide a list of high-confidence ADAR-edited sites within EEF miRNAs, we considered only miRNAs that are derived from *bona fide* miRNA genes (Fromm et al., 2015). Six miRNAs were found to be edited by ADAR enzymes in our data, with two-thirds of them (i.e., 4) edited in the seed region (Figure 8). Given the length of the seed regions (about one-third of the mature miRNA length), this suggested that nucleotides within miRNA seed regions are prone to editing more often than expected by chance (*p* ∼0.08). MiR-376b-3p (at position 6), miR-3099-3p (at position 7), and miR-378a (at position 16) were heavily edited (at least 50% of samples) while miR-381-3p (at position 7) and miR-421-3p (at position 14) were slightly edited (<10%). All the edited sites were detected in both control and KA groups at both time-points. However, no significant differences in the amount of editing were found between groups or time-points.

We also assessed the confidence of these edited sites. All the edited sites have been reported as “confident” in a recent comprehensive analysis profiling ADAR editing (Li et al., 2018). In addition, three of them (miR-411-5p at position 5, miR-376b-3p at position 6, and miR-421-3p at position 14) have been reported in brain (Alon et al., 2012), with comparable editing levels, while miR-411-5p (at position 5) has been reported in plasma-derived exosomes (Nigita et al., 2018). Moreover, they all have preferable sequence motifs for ADARs, which is uridine (U) enriched and guanosine (G) depleted at the nucleotide preceding the edited sites (four U and zero G out of six) and guanosine enriched at the nucleotides following the edited sites (five out of six) (Figure 8). Taken together, these findings indicate that they are very likely true edited sites in EEF-miRNAs.

## Discussion

The present study characterized the effects of experimental status epilepticus on the exosome pathway, miRNA content, and editing. We show EEFs from the mouse hippocampus contain a diverse set of miRNAs that likely reflect contributions from multiple cell types. Status epilepticus caused relatively minor changes to the exosome pathway components but select time-dependent changes to the content, although not editing, of EEF miRNAs. Together, the results provide the first evidence that exosomes and their miRNA content may serve roles in the pathophysiological responses to status epilepticus.

There is growing interest in the role of microvesicles and their miRNA content in the pathophysiology of brain diseases (Levy, 2017; Zhang et al., 2019). The present study provides a comprehensive analysis of the expression of exosome biogenesis pathways in the hippocampus and how these are affected by status epilepticus. Overall, we found that status epilepticus produces changes to transcript levels and proteins associated with both ESCRT-dependent and -independent pathways (Colombo et al., 2014). This was bi-directional, occurred bi-laterally, and was time-dependent, although the scale of the observed changes was modest. Despite overt pathological changes being mainly restricted to the CA3 subfield in this model (Araki et al., 2002; Diviney et al., 2015; Jimenez-Mateos et al., 2015), changes were observed in all major hippocampal subfields. This suggests that seizure activity as well as neuronal damage may be a driver of these transcriptional responses. The most extensive changes were found in the DG subfield, a region that not only displays cell death but also contains the neurogenic zone of the hippocampus. Since new-born and migrating neurons have been reported to produce and communicate using exosomes (Batiz et al., 2015), it is possible that the changes we detected after status epilepticus in this subfield reflect exosome biogenesis responses during the increase in neurogenesis that accompanies status epilepticus in this model (Beamer et al., 2018).

Several of the differentially expressed exosome pathway genes have been previously associated with epilepsy. Alix was reported to be differentially expressed in two epilepsy models and linked to endocytosis (Han et al., 2019) and changes to Rab27a levels were present in human epilepsy studies (Wang et al., 2014). The findings in these reports may, therefore, reflect changes to exosome biogenesis. The ESCRT-independent pathway showed particularly consistent dysregulation after status epilepticus. This included the tetraspanin CD63, which is highly associated with exosomes and involved in cargo selection (van Niel et al., 2011). This may reflect an overall increase in exosomal generation (van Niel et al., 2011) or an increase in CD63-enriched exosome production. Moreover, CD63 changes were minimal on the contralateral side supporting pathophysiological significance as the ipsilateral hippocampus and CA3 subfields are sites of spontaneous seizure generation in the model (Li et al., 2008). Pld2 showed relatively few changes, although a small increase in *Pld2* levels was found within the ipsilateral hippocampus at the 2-week time-point. Changes to Pld2 have been reported after status epilepticus within reactive astrocytes and also DG granule neurons (Kim et al., 2004). Again, this may reflect changes to exosome production since Pld2 levels correlate with the proportion of exosomes released (Laulagnier et al., 2004; Ghossoub et al., 2014).

An unexpected finding in the present study was the restriction of transcriptional changes to the first 24 h after status epilepticus whereas most protein changes were observed in the 2-week samples. This suggests a temporal change between transcriptional and translational mechanisms regulating the exosome pathway. Status epilepticus may drive modest changes to the transcription of exosome biogenesis genes but this abates thereafter. Spontaneous seizures, which likely occurred in mice in the 2-week group, unlike status epilepticus, may not be a strong driver of transcriptional responses of the exosome pathway in the hippocampus. In contrast, changes to proteins associated with exosome biogenesis were mainly found in the 2-week samples, including upregulation of Alix, Tsg101, and Rab27a proteins. The DG again displayed the most differences in the exosome biogenesis components. Notably, neurogenesis-modulating miRNAs such as miR-9, miR-124, and miR-128 are incorporated into exosomes (Huang et al., 2013). The neurogenic niche within the DG may therefore continue to display enhanced exosome production and exosomes may contribute to the aberrant neurogenesis associated with chronic epilepsy. We may speculate that posttranscriptional mechanisms dominate the regulation of the exosome biogenesis pathway in epilepsy, perhaps mediated by the alterations to expression of miRNAs.

The present study includes the first analysis of miRNA content within EEFs derived from the hippocampus after status epilepticus. Using small RNA sequencing we were able to detect levels of known exosome-enriched miRNAs in EEFs prepared from the mouse hippocampus. The number of unique miRNAs detected was high (397 miRNAs shared between the two EEF preparation methods) and bioinformatics analysis indicated contributions from both neurons and glia. This agrees broadly with other reports (Guduric-Fuchs et al., 2012) although some miRNAs appear to be selectively packaged within exosomes according to specific organs or cell-type (Li et al., 2013; Koppers-Lalic et al., 2014). There was incomplete overlap with exosomal miRNAs reported in the ExoCarta database (Mathivanan and Simpson, 2009), suggesting that either that database is incomplete or that a portion of the miRNA recovered using the two techniques here are contaminants. The miRNA content of EEFs from the mouse hippocampus overlaps extensively with other work using brain cells and tissue (Bellingham et al., 2012; Yelamanchili et al., 2015; Harrison et al., 2016; Ebrahimkhani et al., 2017; Raoof et al., 2017). Notably, more than two-thirds of the mouse hippocampal miRNAs we detected are also present in primate brain exosomes, suggesting high species conservation and translational relevance (Yelamanchili et al., 2015).

Exosomes have been proposed to serve pathophysiological and paracrine signaling roles and we detected changes to ∼5% of the detected miRNAs after status epilepticus. This suggests a selective process of altered exosomal miRNA content during and after epileptogenesis. The changes were mainly upregulation and were found for miRNAs of both neuronal as well as glial origin, indicating status epilepticus alters the exosomal miRNA content from diverse cell types. This included changes to miR-21a-3p and -5p (at 24 h) and miR-146a-5p (at 2 weeks). These miRNAs have previously been reported to be upregulated in experimental and human epilepsy (Aronica et al., 2010; Jimenez-Mateos et al., 2011; Risbud and Porter, 2013; Kretschmann et al., 2015). The EEF changes may be reflecting increased loading or overall abundance of exosomal miRNAs or be due to cell-specific changes in EEF production. The most extensive exosomal pathway changes were observed in the DG, where neuronal cells divide, and our pathway enrichment analysis for the targets of these differentially expressed miRNAs showed these targets were most significantly enriched among GO terms associated with the regulation of cell cycle and division processes. This finding corroborates the suggestion that exosome contents are potentially relevant to the regulation of neurogenesis in epilepsy. Further studies are needed to determine whether specific mechanisms selectively adjust miRNA content of exosomes in epilepsy (Koppers-Lalic et al., 2014). While our results agree with other reports on miRNA content of brain exosomes, we did not detect the miRNAs reported to be differentially expressed in blood-derived exosomes from patients with epilepsy (Yan et al., 2017). Technical factors could explain these discrepancies or the miRNAs detected in that study may not have originated from the brain. In contrast, some of the miRNAs differentially expressed in mouse hippocampus EEFs after status epilepticus are also altered in exosomes extracted from the cerebrospinal fluid of patients (Raoof et al., 2017). Different mechanisms may operate between local (brain tissue) release and eventual appearance in biofluids such as CSF. Notably, miR-21-5p showed a high fold change in cerebrospinal fluid samples suggesting it may be trafficked by extracellular vesicles after seizures in humans and sampling miR-21 in exosomes could be diagnostic of status epilepticus or epileptogenesis (Raoof et al., 2017).

The effects, if any, of EEF-enclosed miRNAs on recipient cells are poorly understood. Studies have reported that miRNAs within extracellular vesicles can regulate inflammation and neuronal damage (Lehmann et al., 2012; Yelamanchili et al., 2015; Harrison et al., 2016). Since inflammation is a common pathomechanism in epilepsy (Vezzani et al., 2011), extracellular vesicles such as exosomes could be functionally important as regulators of a pathological process in the present model. Increased expression of miR-21 has been observed in models of neuro-inflammation and neuronal damage (Strickland et al., 2011; Bergman et al., 2013; Risbud and Porter, 2013; Yelamanchili et al., 2015; Harrison et al., 2016). While the specific targets are not known, miR-21 has been shown to regulate neurotrophin-3 mRNA in the hippocampus (Risbud et al., 2011). The *Mef2c* transcript, which encodes a neuronal transcription factor, is also a target of miR-21 (Yelamanchili et al., 2010). Higher exosomal levels of miR-21 have been reported in the injury boundary zone near reactive microglia after traumatic brain injury (Harrison et al., 2016). Studies have also found exosomal miR-21 to regulate neurotoxicity (Yelamanchili et al., 2015). Prion-infected neurons also show increased miR-21 in exosomes *in vitro* (Bellingham et al., 2012). Thus, miR-21 appears to be a strong candidate differentially expressed exosomal miRNA but its upregulation may not be specific for epilepsy. This may have implications for other uses of exosomal miRNAs, such as diagnostic or prognostic biomarkers. Functional studies, selectively targeting miRNAs within exosomes will be needed to resolve whether the amounts of miRNA within exosomes are sufficient to exert biological effects in recipient cells (Chevillet et al., 2014).

The present study also investigated miRNA editing, revealing six highly confident edited sites within miRNAs from hippocampus-derived EEFs. Since editing within the seed region is likely to alter the targets of the miRNAs, these findings suggest editing may alter the target pool of EEF-enclosed miRNAs. Status epilepticus did not notably change the abundance of the editing. This indicates tissue and disease-specific differences in editing of EEF miRNAs (Li et al., 2018). Notably, our brain tissue findings show only one common editing event (miR-411-5p, position 5) with results from a plasma study (Nigita et al., 2018) and, for example, miR-381-3p is edited at position seven in hippocampal exosomes but at position 4 in plasma-derived exosomes. Together, this implies that the select editing events of exosomal miRNAs are regulated by different pathways, which in turn are modulated under different pathophysiological conditions. Further investigation of editing of miRNAs from other cellular functional fractions such as Argonuate-2-bound miRNAs, which are functionally active, in hippocampal tissues may reveal distinct editing events that are exclusive for EEF or Argonuate-2-bound miRNAs as well as new epilepsy-associated editing patterns of miRNAs.

In summary, the present study provides evidence that status epilepticus produces moderate, select changes to the exosome biogenesis pathway in the mouse hippocampus. We show that EEFs from the hippocampus contain diverse miRNAs and that status epilepticus produces select changes, particularly to those of glial origin or relating to DG functions, potentially reflecting ongoing pathophysiological responses during epileptogenesis or maintenance of the chronic epileptic state. Future studies will be needed to determine whether exosomal miRNAs within biofluids have a mechanistic link to central pathophysiology, explore inter-cellular exchange of miRNAs via exosomes, and whether this has potential therapeutic relevance in epilepsy.

## Data Availability Statement

The datasets generated for this study can be found in the GSE136695.

## Ethics Statement

The animal study was reviewed and approved by the Research Ethics Committee of the Royal College of Surgeons in Ireland (REC #842 and #1133) under license from the Health Products Regulatory Authority (AE19127/I084, AE19127/I089, and AE19127/I152), Dublin, Ireland.

## Author Contributions

AB performed the exosome preparation and analyses. TH and MD performed the animal studies. NN and CM performed the RNA-seq analysis. EL and AS-R performed the histological and husbandry support. GB performed the molecular analyses. NC and NN performed the pathway and target analyses. BC performed the imaging. DH designed the study. AB and DH wrote the manuscript. All the authors reviewed and approved the final version of the manuscript.

## Conflict of Interest

The authors declare that the research was conducted in the absence of any commercial or financial relationships that could be construed as a potential conflict of interest.

## Footnotes

1 https://github.com/NgocTNguyen/mirna-differential-editing-analysis
