# Supplementary material for: Altered Biogenesis and MicroRNA Content of Hippocampal Exosomes Following Experimental Status Epilepticus
Source: Front Neurosci. 2020 Jan 17;13:1404. doi: 10.3389/fnins.2019.01404 (PMC6978807; doi:10.3389/fnins.2019.01404)
Supplement: Supplementary file 1 [file Data_Sheet_1.PDF]

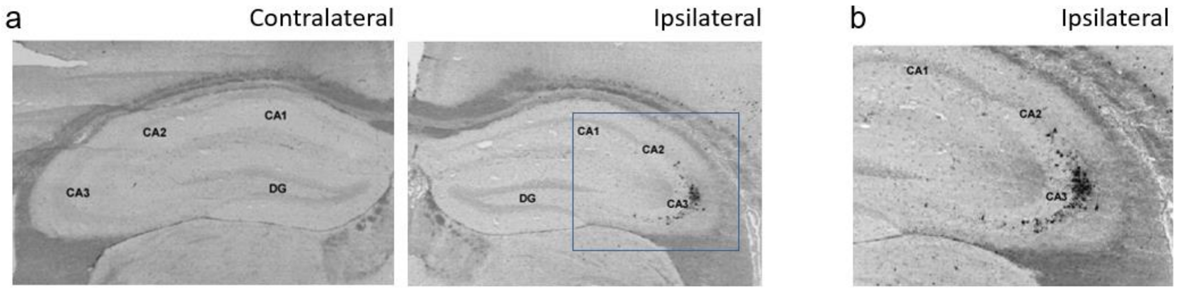

Supplementary Figure 1: *Histology of the seizure-damaged hippocampus in the intraamygdala kainic acid model of status epilepticus*

Figure shows representative brain tissue sections (12 µm coronal) stained by the FluoroJade B technique for irreversible cell death. (a) contralateral and ipsilateral side of a representative section obtained 24 h after status epilepticus and (b) high-magnification of the ipsilateral side showing the CA3-predominant lesion. Image is greyscale inverted thereby showing Fluorojade B-positive cells in black.
