# Supplementary material for: Altered Biogenesis and MicroRNA Content of Hippocampal Exosomes Following Experimental Status Epilepticus
Source: Front Neurosci. 2020 Jan 17;13:1404. doi: 10.3389/fnins.2019.01404 (PMC6978807; doi:10.3389/fnins.2019.01404)
Supplement: Supplementary file 2 [file Data_Sheet_2.PDF]

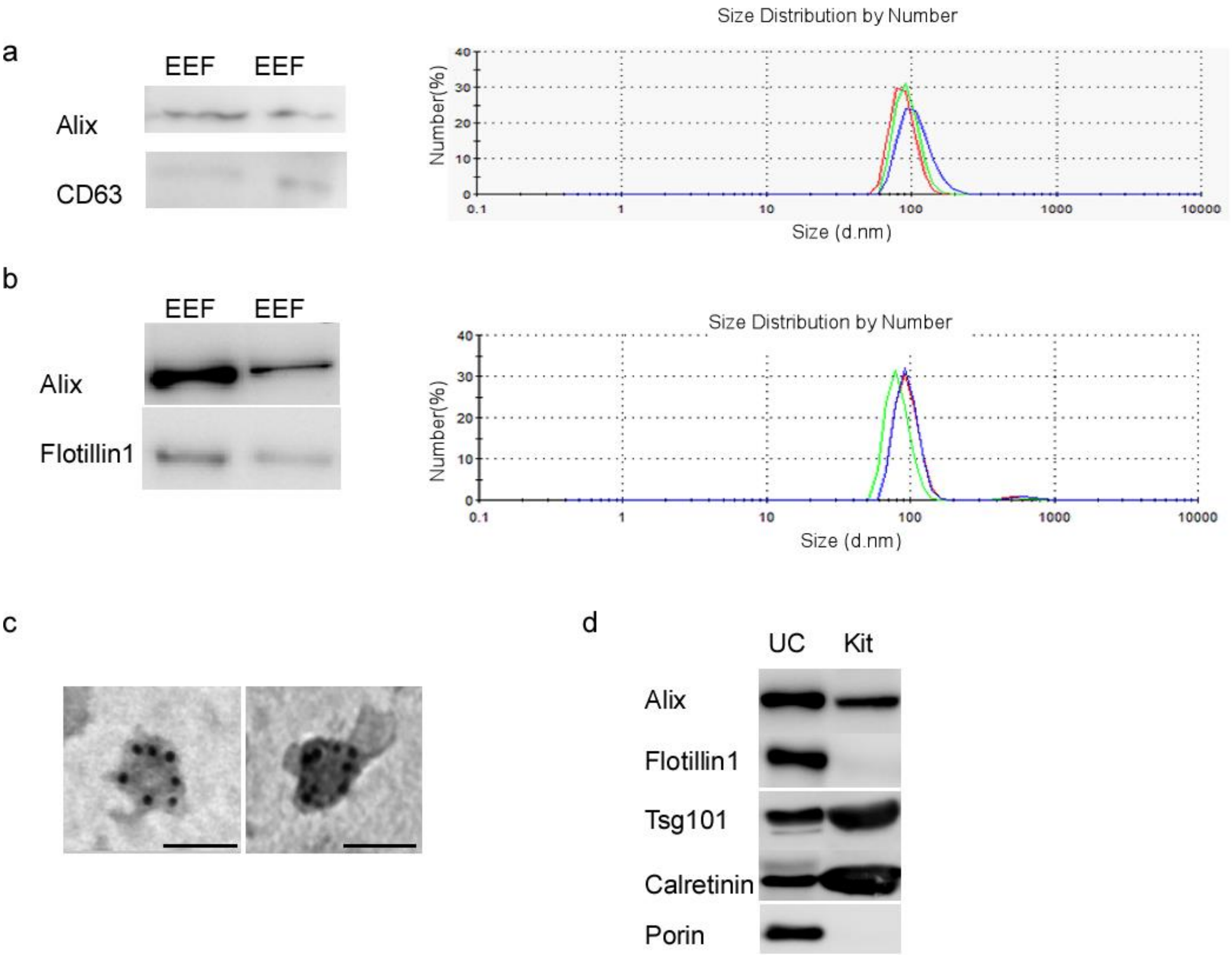

Supplementary Figure 2: *Characterisation of exosomes derived from mouse brain*.

Figure shows exosome features for brain samples processed using either (a) Kit-based or (b) ultracentrifugation (UC) method; expression of exosome-associated proteins and representative zetasizer results using dynamic light scattering to measure size of obtained exosome-enriched fractions (EEF). (c) Immuno-gold staining for CD63 on isolations from mouse brain as seen under transmission electron microscope with uranyl acetate staining, scale bar: 100nm, direct magnification of 100,000X. (d) Comparison of UC and Kit-derived exosome-enriched fractions. Representative immunoblots show Alix, Flotillin1 and Tsg101 in exosome-enriched fractions from mouse brain as well as contaminating proteins Calretinin (endoplasmic reticulum marker) and Porin (mitochondrial marker).
